# PERK–KIPK–KCBP signalling negatively regulates root growth in *Arabidopsis thaliana*

**DOI:** 10.1093/jxb/eru390

**Published:** 2014-09-26

**Authors:** Tania V. Humphrey, Katrina E. Haasen, May Grace Aldea-Brydges, He Sun, Yara Zayed, Emily Indriolo, Daphne R. Goring

**Affiliations:** Department of Cell & Systems Biology, University of Toronto, Toronto, Canada M5S 3B2

**Keywords:** Proline-rich extensin-like receptor-like kinase, KCBP-interacting protein kinase, kinesin-like calmodulin-binding protein, root growth, signalling, sucrose.

## Abstract

The *Arabidopsis* proline-rich, extensin-like receptor-like kinases (PERKs) are a small group of receptor-like kinases that are thought to act as sensors at the cell wall through their predicted proline-rich extracellular domains. In this study, we focused on the characterization of a subclade of three *Arabidopsis* predicted *PERK* genes, *PERK8*, -*9*, and -*10*, for which no functions were known. Yeast two-hybrid interaction studies were conducted with the PERK8,- 9, and -10 cytosolic kinase domains, and two members of the *Arabidopsis* AGC VIII kinase family were identified as interacting proteins: AGC1-9 and the closely related kinesin-like calmodulin-binding protein (KCBP)-interacting protein kinase (KIPK). As KIPK has been identified previously as an interactor of KCBP, these interactions were also examined further and confirmed in this study. Finally, T-DNA mutants for each gene were screened for altered phenotypes under different conditions, and from these screens, a role for the *PERK*, *KIPK*, and *KCBP* genes in negatively regulating root growth was uncovered.

## Summary

We established an interaction network among three *Arabidopsis* PERK receptor-like kinases (8, 9, and 10), two AGC VIII kinases (AGC1-9 and KIPK) and KCBP with a role in negatively regulating root growth.

## Introduction

Plant receptor-like kinases make up the largest class of kinases in plant genomes, with over 600 predicted members in *Arabidopsis*, and have a large range of functions from plant development to plant–microbe interactions and abiotic stress responses (reviewed by Gish and Clark, 2011; Antolin-Llovera *et al.*, 2012; Osakabe *et al.*, 2013). One predicted subclass of the plant receptor-like kinases is the proline-rich, extensin-like receptor-like kinases (PERKs) (Silva and Goring, 2002). With their predicted proline-rich, extensin-like extracellular domains, PERKs are thought to be part of a group of proteins that act as sensors/receptors at the cell wall. These sensors/receptors, for example, may monitor changes to the cell wall during cell expansion in plant growth, or during plant exposure to abiotic/biotic stresses, and activate associated cellular responses (reviewed by Humphrey *et al.*, 2007; Steinwand and Kieber, 2010; Doblin *et al.*, 2014).

The first PERK member to be characterized was *Brassica napus PERK1*, which was found to be expressed in a number of different tissues as well as rapidly induced by wounding (Silva and Goring, 2002). The expression patterns of the 15-member *Arabidopsis PERK* gene family were subsequently characterized, and several were found to be broadly expressed (*PERK1*, -*2*, -*3*, -*8*, -*9*, -*10*, -*14*, and -*15*), while others showed high expression in specific tissues such as the root (*PERK13*) and pollen (*PERK4*, -*5*, -*6*, -*7*, -*11*, and -*12*) (Nakhamchik *et al.*, 2004). PERKs are predicted to be membrane localized with a cluster of positively charged amino acids next to the transmembrane domain orienting the proline-rich domains on the external face of the plasma membrane and the kinase domain internally (Silva and Goring, 2002; Nakhamchik *et al.*, 2004). Consistent with this, BnPERK1 was found to have a serine/threonine kinase activity *in vitro*, and when fused to green fluorescent protein (BnPERK1:GFP) was localized to the plasma membrane when transiently expressed in onion cells (Silva and Goring, 2002). In addition, a BnPERK1:haemagglutinin fusion expressed in *Arabidopsis* was enriched in the plasma membrane fraction, following aqueous two-phase partitioning (Haffani *et al.*, 2006). *Arabidopsis* PERK4 has also been shown to have kinase activity, and a GFP:PERK4 fusion was localized to the plasma membrane and appeared to be associated with the cell wall (Bai *et al.*, 2009).

Defining clear roles for PERK genes during plant growth and development has been challenging, and the ectopic expression or suppression of *PERK* genes in *Arabidopsis* can lead to complex changes in growth and organ development. When *BnPERK1* was expressed ectopically in *Arabidopsis*, there were several altered traits including an increased number of lateral shoots, and an increased number of ovules per pistil and seeds per silique (Haffani *et al.*, 2006). Ectopic callose and cellulose deposits were also detected in these transgenic plants suggesting cell wall alterations. The antisense expression of *BnPERK1* in *Arabidopsis* led to the suppression of tandemly linked *Arabidopsis PERK1* and -*3* genes, and resulted in reduced apical dominance and abnormal floral organs (Haffani *et al.*, 2006). In addition, hypocotyls from dark-grown, *PERK1*- and -*3*-supressed seedlings were longer relative to the wild-type *Arabidopsis* Columbia ecotype (Col-0), while seedlings with ectopic *BnPERK1* expression displayed shorter hypocotyls (Haffani *et al.*, 2006). Complex traits were similarly found with the ectopic expression of *Arabidopsis PERK12* in the activation-tagged mutant *inflorescence growth inhibitor1* (*igi1*). Hwang *et al.* (2010) identified *igi1* in a screen for increased shoot-branching mutants, and *igi1* displayed a dwarf phenotype with increased axillary branching and abnormal floral organs. This phenotype resulted from the T-DNA carrying four enhancers inserting in the 5′ end of the *PERK12* gene and driving strong ectopic expression of this normally pollen-specific gene (Hwang *et al.*, 2010).

More specific functions have been uncovered for the *Arabidopsis PERK4* and -*13* genes. *PERK13* (also called *RHS10*) was identified in a screen for *Arabidopsis* genes displaying root hair cell-specific expression (Won *et al.*, 2009). *PERK13/RHS10 Promoter:GFP* plants displayed GFP fluorescence in the root hairs, and a *perk13/rhs10* T-DNA insertion mutant had longer root hairs compared with wild-type. Using a strong root hair promoter, Won *et al.* (2009) also overexpressed *PERK13/RHS10* in root hairs, and this resulted in an inhibition of root hair elongation and shorter root hairs. In the case of *PERK4*, *perk4* T-DNA mutants were found to be less sensitive to the inhibitory effects of abscisic acid (ABA) and had increased germination rates and root length in the presence of ABA compared with wild-type Col-0 roots (Bai *et al.*, 2009). Under these conditions, the increased root length appeared to be due to increased cell elongation relative to wild-type Col-0 seedlings. Finally, there was reduced ABA-mediated calcium channel activity in the *perk4* mutant roots relative to Col-0, suggesting a role for calcium fluxes in the ABA-mediated inhibition of root elongation (Bai *et al.*, 2009).

While nothing is known about downstream interacting/signalling proteins for PERKs, the kinase domain from *Arabidopsis* PERK1 was isolated previously as an interactor for the nuclear shuttle protein (NSP) from cabbage leaf curl virus (Florentino *et al.*, 2006). NSP is needed to transport the viral DNA from the nucleus to the cytoplasm, and NSP functions with the movement protein in spreading the viral DNA from cell to cell. *Arabidopsis* PERK1 (also called NSP-associated kinase or NsAK) was found to phosphorylate NSP, and a *perk1/nsak* T-DNA insertion mutant had increased tolerance to cabbage leaf curl virus infection, with a delay in symptom development, suggesting that PERK1/NsAK positively regulates NSP function, perhaps playing a role in cell-to-cell movement (Florentino *et al.*, 2006).

In this study, we focused on a subclade of three *Arabidopsis PERK* genes, *PERK8*, -*9*, -*10*, which show widespread expression in *Arabidopsis* (Silva and Goring, 2002; Nakhamchik *et al.*, 2004). Two different approaches were taken simultaneously to characterize these genes: (i) a yeast two-hybrid screen was conducted with the PERK10 cytosolic domain to identify interacting proteins; and (ii) T-DNA knockout mutants were screened for altered root growth under different conditions. Interactors of PERK8, -9, and -10 were identified as AGC1-9 and the closely related kinesin-like calmodulin-binding protein (KCBP)-interacting protein kinase (KIPK), members of the *Arabidopsis* AGC VIII kinase family (Zegzouti *et al.*, 2006; Rademacher and Offringa, 2012). KIPK was previously identified as an interactor of KCBP (Day *et al.*, 2000), and this interaction was examined further in this study. Finally, using T-DNA mutants, we uncovered a role for the *PERK*, *KIPK*, and *KCBP* genes in negatively regulating root growth.

## Materials and methods

### Yeast two-hybrid screen

For the yeast two-hybrid library, random-primed cDNA was synthesized from flower bud mRNA and inserted into pGADT7-Rec (GAL4 activation domain) using the Matchmaker library construction and screening kit (BD Biosciences Clontech). For the PERK constructs, the entire cytosolic kinase domains (just after the transmembrane domains) were PCR amplified and cloned as fusions to the GAL4 DNA-binding domain into pGBKT7 (BD Biosciences Clontech). PERK10 was used to screen the yeast two-hybrid library according to the kit instructions. Following the screening of positive colonies on quadruple dropout plates (SD–Ade–His–Leu–Trp), plate assays were conducted on the remaining positive colonies to test for α-galactosidase activity (*MEL1* reporter) in the presence of 5-bromo-4-chloro-3-indolyl-α-d-galactopyranoside (X-α-Gal), or filter lifts were performed to test β-galactosidase activity (*lacZ* reporter) in the presence of 5-bromo-4-chloro-3-indolyl-β-d-galactoside (X-Gal). The plasmids from these positive yeast colonies were isolated directly (Ding *et al.*, 2003), retested for interactions with PERK10, and sequenced (Genome Quebec sequencing facility). Further screening was conducted to remove non-specific interactors, and KIPK2 was chosen for further analysis. The KIPK2 clones from the library screen contained the region of aa 85–324 or 85–355.

For further yeast two-hybrid interaction testing, the full-length KIPK2 cDNA was PCR amplified and cloned into pGEM-T Easy (Promega) while the full-length KIPK1 cDNA was obtained as a pUni clone (U22072) (Yamada *et al.*, 2003) from the Arabidopsis Biological Resource Center (ABRC). Different segments from the KIPK1 and KIPK2 cDNA were then transferred to create an in-frame fusion with the GAL4 activation domain in pGADT7-Rec. For KIPK1, the KIPK1_84–312_ segment was PCR amplified, while internal restriction sites were used for the other constructs: KIPK1_84–582_ (*Cfr*10I–*Sma*I), KIPK1_303–582_ (*Ssp*I–*Sma*I), and KIPK1_303–934_ (*Ssp*I–*Sal*I in vector). For KIPK2, one of the original yeast two-hybrid clones was KIPK2_85–324_, and internal restriction sites were used for the other constructs: KIPK2_85–624_ (start of KIPK2_85–324_–*Fsp*I), KIPK2_277–624_ (*Nde*I–*Fsp*I), and KIPK2_277–948_ (*Nde*I–*Eco*R1 in vector). For KCBP, the pAS1CYH2/N-terminal KCBP plasmid containing aa 12–923 was obtained from Dr Anireddy Reddy. KCBP_12–923_ was then transferred from this plasmid as an *Nco*I fragment to construct an in-frame fusion to the GAL4 DNA-binding domain in pGBKT7. For the smaller KCBP segments, internal restriction sites were used: KCBP_12–294_ (*Nco*I–*Nde*lI), KCBP_12–496_ (*Nco*I–*Eco*RI), KCBP_12–622_ (*Nco*I–*Psp*1406I), and KCBP_465–923_ (*Eco*RV*Nco*I). Pairwise combinations of the pGBKT7- and pGADT7-based plasmids were transformed into the yeast Y187 strain and selected for on SD–Leu–Trp plates. Filter lifts were performed on the transformed colonies to test for β-galactosidase activity in the presence of X-Gal (producing a blue colour for positive interactions).

### Plant material and growth conditions

The *Arabidopsis* SALK T-DNA insertion lines (Alonso *et al.*, 2003) were obtained from the ABRC at Ohio State University. The *Arabidopsis* GABI-Kat T-DNA insertion line for KIPK2 was obtained from the GABI-Kat collection at the Max Planck Institute of Plant Breeding Research (Rosso *et al.*, 2003). Wild-type *Arabidopsis thaliana* (Col-0) and transgenic seeds were surface sterilized with a 1:1 (95% ethanol:3% H_2_O_2_) solution. Seeds were rinsed three to five times with sterile distilled water. All seeds were placed at 4 °C in the dark in either a 0.01% agar solution or on the medium plates themselves to synchronize germination. Seeds were germinated on ½ Murashige and Skoog salts (½ MS) (Cassion Laboratories) and 0.8% (w/v) agar (Phytoblend; Cassion Laboratories) at pH 5.8, unless otherwise stated. Long-day growth chamber conditions consisted of a 16h light/8h dark photoperiod at 22 °C. Constant light conditions were a 24h light cycle at 22 °C. Plants were grown on autoclaved potting soil (Premiere ProMix) and fertilized with a 20-20-20 mixture (Plant Pro Fertilizer).

### T-DNA insertion line screens and reverse transcription (RT)-PCR analyses

Combinations of T-DNA and gene-specific PCR-based screens on genomic DNA were used to identify homozygous T-DNA insertion lines. These lines were then crossed to generate the multiple *perk*, *kipk*, and *kcbp* homozygous T-DNA insertion mutants. The homozygous plants were confirmed as knockouts by conducting RT-PCR using RNA extracted from flower buds (except for KCBP where seedling RNA was used). For expression profiling of *PERK8*, *PERK9*, *PERK10*, *KIPK1*, *KIPK2*, and *KCBP* genes, RNA was extracted from 2-week-old seedling tissue (whole seedlings, rosettes, roots, or hypocotyls). The PCR primers used are listed in Supplementary Table S1 at *JXB* online.

### Sucrose response assays

Seeds were sterilized as described above and plated on Phytoblend medium plates. After 48h at 4 °C in the dark, the plates were placed vertically in a 16h light/8h dark photoperiod at 22 °C for 7 d. The 7-d-old seedlings were then transferred to sucrose treatment plates (Phytoblend) containing 0, 0.5, or 4.5% sucrose, lining up the root tips, and placed at 22 °C under 24h light (under approx. 80–110 µmol m^–2^ sec^–1^). After 7 d, digital photographs were taken, and the new root growth (since transfer) was measured from the digital images using ImageJ software (Abramoff *et al.*, 2004).

### *DEX::PERK10* transgenic *Arabidopsis*

The full-length PERK10 cDNA was inserted next to the dexamethasone (DEX)-inducible promoter in the pTA7002 plant transformation vector (Aoyama and Chua, 1997) and transformed into Col-0, the *perk8-1,9-1,10-1* triple mutant, the *kipk1-1,2-1* double mutant, and the *kcbp-1* mutant using the floral dip method (Clough and Bent, 1998). Primary transformants were identified by hygromycin B selection (25mg l^–1^) on ½ MS agar plates and then transferred to soil. Seeds were then collected from each individual primary transformant for further testing. For the DEX plate germination assays, seeds were sterilized, sown on ½ MS agar plates with 10 µM DEX, and stratified at 4 °C for 3 d in the dark. The plates were then transferred to a 16h light/8h dark photoperiod at 22 °C, and after 5 d, the phenotypes of the germinated seedlings were scored. To confirm *PERK10* expression with DEX treatment, RNA was extracted from untreated and treated seedlings for RT-PCR analysis. For phloroglucinol staining of lignin, samples were placed in a 1% phloroglucinol/HCl solution, shaken gently for 10min, and then mounted in a 50% glycerol/6M HCl solution and observed under bright-field illumination (Rogers *et al.*, 2005). For detection of callose, roots were placed in a decolorized aniline blue solution (Sigma; resuspended at 0.1mg ml^–1^ in phosphate buffer, pH 9.0).

## Results

### Yeast two-hybrid screen for PERK10-interacting proteins

**Fig. 1.**
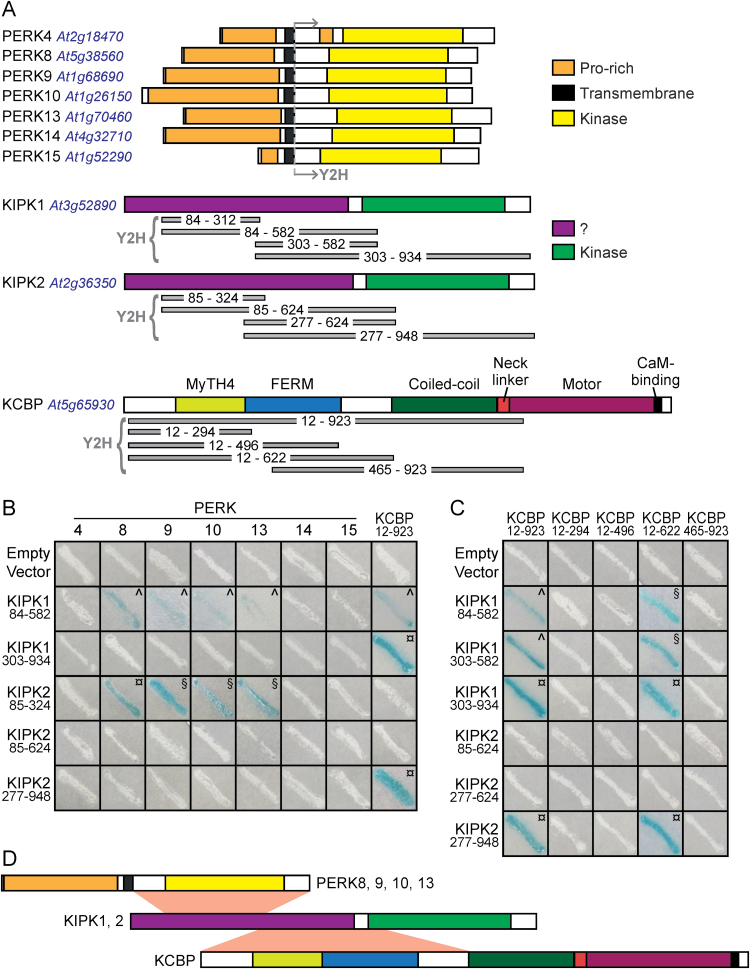
Yeast two-hybrid analyses for protein–protein interactions between the PERK, KIPK, and KCBP proteins. (A) Predicted protein domains and segments tested in the yeast two-hybrid system. The predicted PERK domains are from Nakhamchik *et al.* (2004), the KIPK1 and -2 kinase domains were predicted from The Arabidopsis Information Resource (TAIR) and SMART (Schultz *et al.*, 1998), and the KCBP domain organization is from Abdel-Ghany *et al.* (2005). The regions tested in the yeast two-hybrid system are shown in grey beneath each gene. (B) Yeast two-hybrid interactions between the PERK and KIPK proteins. The cytosolic domains from PERK4, -8, -9, -10, -13, -14, and -15 were tested for interactions against different subdomains of KIPK1 and KIPK2. Both PERK1 and KIPK1_84–312_ were initially included in the screen but may have been toxic, as yeast colonies could not be recovered for either construct. (C) Yeast two-hybrid interactions between the KIPK and KCBP proteins. Different subdomains of KIPK1 and KIPK2 were tested for interactions with different subdomains of KCBP. For both (B) and (C), yeast Y187 cells were transformed with the indicated constructs, and positive interactions were scored by the development of a blue colour (from activation of the *lacZ* reporter). Symbols represent time for the blue colour to develop: ¤,Less than 1h; §, 1–3h; ^, more than 3h. The negative interactions (white streaks) were monitored for up to 24h (with the exception of PERK8 and -9 transformants, which were left for shorter incubation times due to some background activity). (D) Schematic of proposed minimal interaction domains between the PERK, KIPK, and KCBP proteins.

Both BnPERK1 and AtPERK4 have been shown to localize to the plasma membrane (Silva and Goring, 2002; Haffani *et al.*, 2006; Bai *et al.*, 2009), and similarly, PERK8, -9, and -10 are predicted to be transmembrane proteins (Fig. 1A; Nakhamchik *et al.*, 2004). Thus, to search for potential downstream signalling proteins for PERK8, -9, and -10, only the cytosolic domains were used in the yeast two-hybrid system. These constructs were first tested for background activity, and from this, PERK10 was chosen for the full-scale screen of a yeast two-hybrid library constructed of random-primed cDNA from *Arabidopsis* flower bud mRNA. Among the positive clones sequenced from this screen was a predicted member of the *Arabidopsis* AGC VIII kinase family (AGC1-9; Zegzouti *et al.*, 2006; Rademacher and Offringa, 2012), which was most closely related to the previously characterized KIPK (Day *et al.*, 2000; Zegzouti *et al.*, 2006). KIPK was renamed KIPK1, and AGC1-9 was renamed KIPK2 (Fig. 1A). Two different KIPK2 clones were identified in this yeast two-hybrid screen and both were partial cDNAs covering aa 85–324 and 85–355, respectively.

**Fig. 2.**
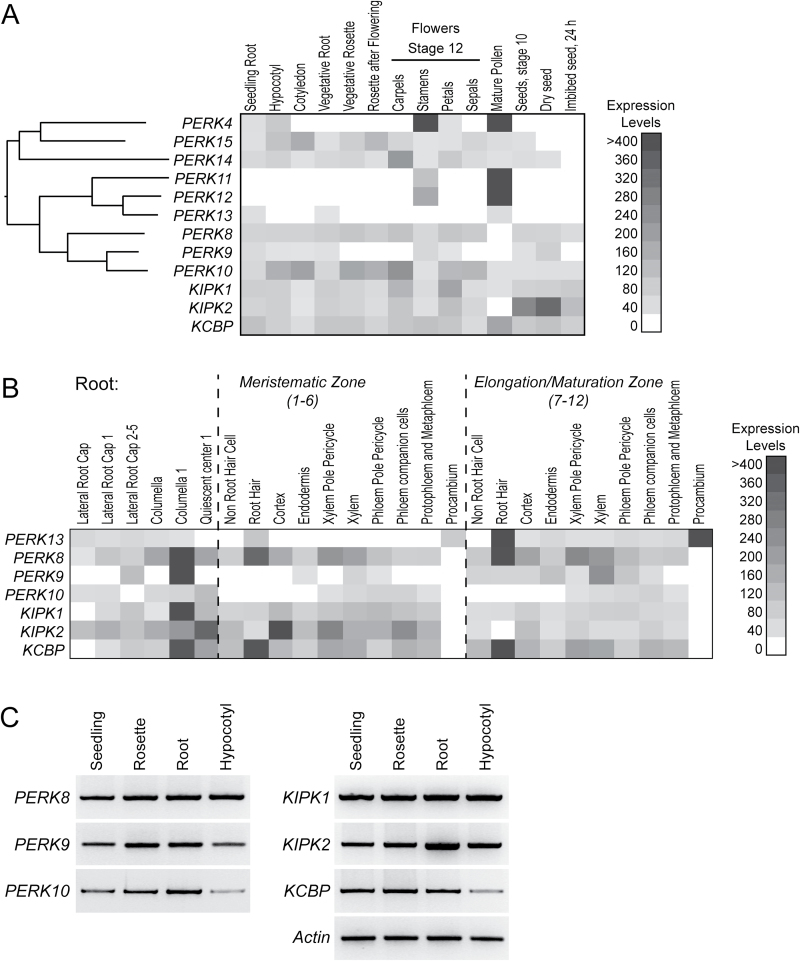
Expression profiles for the *PERK*, *KIPK*, and *KCBP* genes. (A) Heatmap of the microarray expression profiles for the *PERK*, *KIPK*, and *KCBP* genes in datasets for different tissues (Schmid *et al.*, 2005) and seeds (Nakabayashi *et al.*, 2005). Shown next to the PERKs is a protein distance phylogeny built using the cytosolic protein domains (Mobyle, http://mobyle.pasteur.fr) to illustrate the relationships among these family members (Nakhamchik *et al.*, 2004). (B) Heatmap of the microarray expression profiles for the *PERK*, *KIPK*, and *KCBP* genes in the different root tissues dataset (Brady *et al.*, 2007). E-northern analyses of the public microarray datasets were performed through the BioArray Resource (Winter *et al.*, 2007). (C) RT-PCR analysis of *PERK*, *KIPK*, and *KCBP* expression in 2-week-old seedlings. RNA was extracted from whole seedlings, rosettes, roots, and hypocotyls. Actin was used as a positive control for expression in all tissues.

The full-length KIPK2 protein was predicted to be 948 aa with the catalytic domain of a serine/threonine protein kinase predicted to be from aa 559 to 898 (Fig. 1A). KIPK2 and KIPK1 shared 76% amino acid sequence homology (alignment shown in Supplementary Fig. S1 at *JXB* online.). In addition to the kinase domains, KIPK1 and -2 shared a highly similar N-terminal domain that was enriched for serine (Fig. 1A and Supplementary Fig. S1). The specificity of the KIPK2 interaction was tested against a series of PERKs (PERK4, -8, -9, -10, -13, -14, and -15), and only PERK8, -9, -10, and -13 were found to interact with the library clone, KIPK2_84–324_ (Fig. 1B). Of the PERKs tested, PERK8, -9, -10, and -13 were the most closely related to each other (Fig. 2A; Nakhamchik *et al.*, 2004). Despite the high sequence similarity of KIPK1 to KIPK2, it was not isolated in the yeast two-hybrid screen, and so a similarly small KIPK1 construct was designed and tested for interactions with PERKs. However, the KIPK1_84–312_ construct, which covered the same interacting region in KIPK2_84–324_, appeared to be toxic in yeast, as colonies could not be recovered. When a larger segment covering the N-terminal region, KIPK1_84–582_, was tested, it interacted with PERK8, -9, -10, and -13 (Fig. 1B). Interestingly, when a KIPK2 fragment encompassing the same region was tested, KIPK2_84–624_, no interaction could be observed, which may explain why only small partial cDNAs (encoding KIPK2_84–324_ and KIPK2_84–355_) were isolated from the original yeast two-hybrid screen. It is possible that the larger KIPK2 fragment had some type of inhibitory activity associated with it. Finally, constructs lacking a portion of the N-terminal region, KIPK1_303–934_ and KIPK2_277–948_, did not show any interactions with the PERKs (Fig. 1A, B).

KIPK1 was first identified as an interactor for KCBP by Day *et al.* (2000), who used KCBP_12–923_ to screen an *Arabidopsis* yeast two-hybrid library and pulled out KIPK1_148–934_ as an interactor. When subclones of KIPK1 were further tested, KIPK1_513–934_ was found to interact with KCBP_12–923_ while KIPK1_190–537_ did not (Day *et al.*, 2000). KCBP, which belongs to the kinesin-14 family, is unique in its domain organization and is encoded by a single-copy gene in *Arabidopsis* (Fig. 1A) (Reddy *et al.*, 1996; Narasimhulu *et al.*, 1997; Abdel-Ghany *et al.*, 2005). For testing KCBP interactions in our system, a series of KIPK1 and -2 constructs were tested against KCBP_12–923_, and interactions were observed for both KIPK1_303–934_ and KIPK2_277–948_ (Fig. 1A, B). KIPK1_303–934_ and KIPK2_277–948_ interactions were specific to KCBP, confirming that the N-terminal region is needed for interactions with PERKs (e.g. KIPK2_84–324_; Fig. 1A, B). A series of smaller constructs was made to further define the regions of interactions. Both KIPK1_84–582_ and KIPK1_303–582_ interacted with KCBP_12–923_ and the smaller KCBP_12–622_ construct (Fig. 1A, C). KCBP_12–923_ and KCBP_12–622_ only interacted with KIPK2 when the kinase domain was present (KIPK2_277–948_; Fig. 1C). Similar to what was seen for the PERK kinases, the smaller KIPK2 constructs (KIPK2_85–624_ and KIPK2_277–624_) failed to show any detectable interaction with KCBP. In an attempt to further delineate which region of KCBP_12–622_ was interacting with KIPK1 and -2, three other constructs were tested, KCBP_12–294_, KCBP_12–496_, and KCBP_496–923_, but no interactions were detected (Fig. 1C). Altogether from these results, we propose a model where the proximal N-terminal region in KIPK1 and -2 mediates interactions with the PERK kinase domains while the distal N-terminal residues in KIPK1 and -2 are required for interactions with the N-terminal half of KCBP (Fig. 1D).

### Phenotypic screening of *perk*, *kipk*, and *kcbp* T-DNA insertion mutants

With the discovery of the PERK–KIPK–KCBP interactions, the next step was to uncover where this potential signalling pathway might be functioning *in planta*. We focused on PERK8, -9, and -10 as they were the most closely related and were more broadly expressed in *Arabidopsis* (Fig. 2) (Nakhamchik *et al.*, 2004). While PERK13 also interacted with KIPK1 and -2, it had a more specific expression profile (Fig. 2A, B) (Nakhamchik *et al.*, 2004) and mutant phenotype (root hairs; Won *et al.*, 2009), and was most closely related to the pollen-specific PERK11 and -12 (Fig. 2A) (Nakhamchik *et al.*, 2004). Similar to *PERK8*, -*9*, and -*10*, a survey of the public microarray datasets shows that *KIPK1*, *KIPK2*, and *KCBP* were expressed across a range of *Arabidopsis* tissues (Fig. 2A). Overlapping expression profiles for the *PERK(8,9,10)–KIPK(1,2)–KCBP* set were detected in the microarray datasets for the different seedling tissues, the vegetative root, some of the floral tissues, and seeds (Fig. 2A). RT-PCR also confirmed expression of the *PERK(8,9,10)*, *KIPK(1,2)*, and *KCBP* genes in whole seedlings, rosettes, roots and hypocotyls (Fig. 2C).

**Fig. 3.**
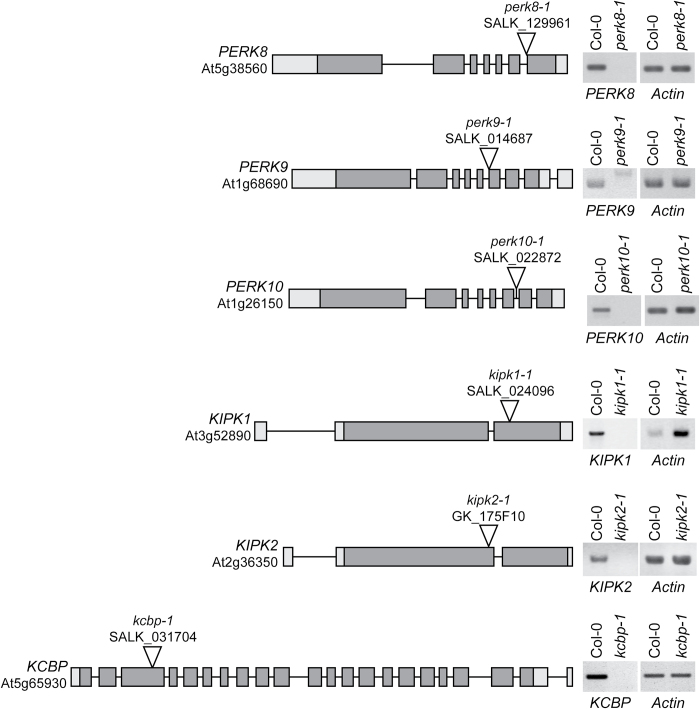
T-DNA insertion sites for the *PERK*, *KIPK*, and *KCBP* genes. On the left, schematics display the *Arabidopsis* SALK T-DNA insertion lines for the *PERK8*, *PERK9*, *PERK10*, *KIPK1*, and *KCBP* genes (Alonso *et al.*, 2003) and the GABI-Kat T-DNA insertion line for *KIPK2* (Rosso *et al.*, 2003). On the right, RT-PCR analyses using gene-specific primers show the loss of gene-specific transcripts in each homozygous mutant, indicating that each T-DNA insertion is a knockout line. Actin primers were used as positive controls for each sample. T-DNA insertion sites were obtained from the SIGnAL website (http://signal.salk.edu), and gene models were obtained from The Arabidopsis Information Resource on 1 June, 2011 (http://www.arabidopsis.org) (Rhee *et al.*, 2003).

*KCBP* is the only gene in the *PERK(8,9,10)–KIPK(1,2)–KCBP* set with a known function in *Arabidopsis*; that is, in trichome morphogenesis. The *zwichel* (*zwi*) mutant, identified in a screen for trichome mutants, was determined to be a loss-of-function *kcbp* mutant and displayed defects in trichome cell expansion resulting in shorter stalks and reduced branching (Oppenheimer *et al.*, 1997). To search for overlapping *in planta* functions for the *PERK(8,9,10)–KIPK(1,2)–KCBP* set, loss-of-function homozygous T-DNA mutants were identified for all six genes (Fig. 3). The mutants were also crossed to form multiple gene knockout mutants and screened for altered phenotypes. As expected, *kcbp-1* displayed the reduced trichome branching phenotype reported previously for the *zwi*/*kcbp* mutant (Oppenheimer *et al.*, 1997). Any mutant combinations with *kcbp-1* also showed the reduced trichome branching: the *perk8-1,9-1,10-1,kcbp-1* quadruple mutant and *kipk1-1,2-1,kcbp-1* triple mutant (Supplementary Fig. S2 at *JXB* online.). However, the trichomes were normal in appearance for the *perk8-1,9-1,10-1* triple mutant and the *kipk1-1,2-1* double mutant (Supplementary Fig. S2), suggesting that these proteins did not participate in the regulation of KCBP during trichome formation. In addition, plants from all these mutant combinations did not display any detectable growth or floral defects when grown on soil (Supplementary Fig. S2).

**Fig. 4.**
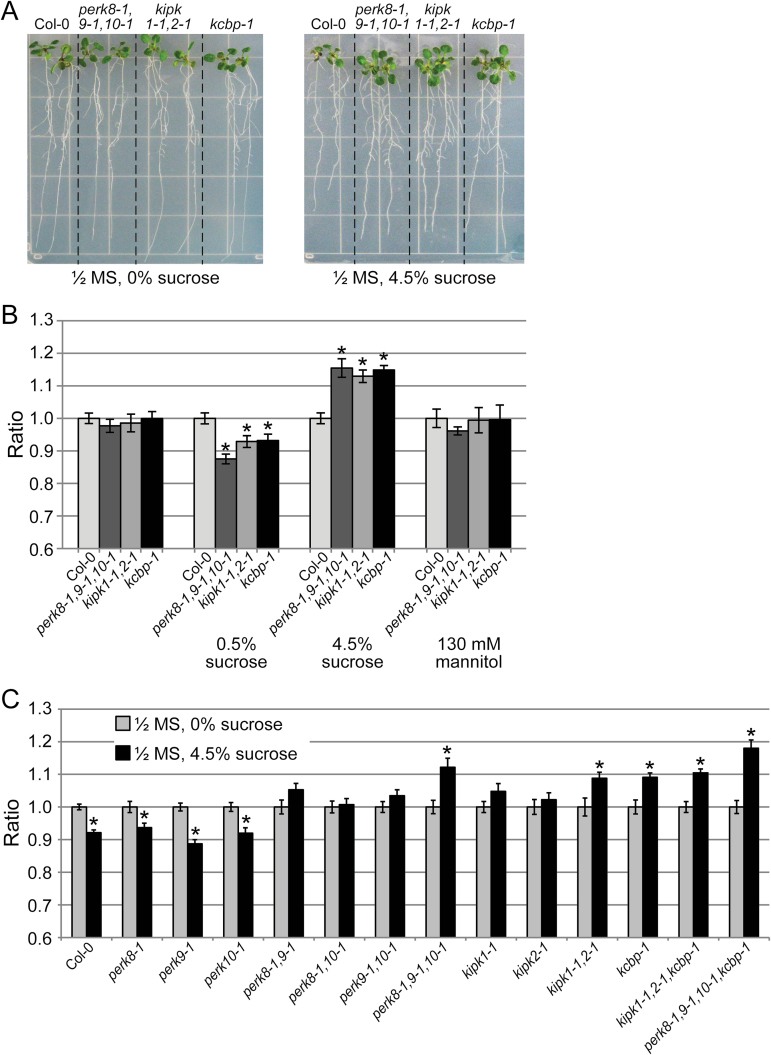
Root growth of the different *perk*, *kipk*, and *kcbp* mutants under high sucrose. (A) Images showing seedling roots for Col-0, the *perk8-1,9-1,10-1* triple mutant, the *kipk1-1,2-1* double mutant, and the *kcbp-1* mutant, grown on ½ MS plates with 0 or 4.5% sucrose under 24h light. (B) Graph showing the ratios of root length at d 7 on different medium conditions, under 24h light, for Col-0, the *perk8-1,9-1,10-1* triple mutant, the *kipk1-1,2-1* double mutant, and the *kcbp-1* mutant. Ratios are relative to Col-0 growth under each condition. Asterisks represent statistically significant differences (*P*<0.05) when compared with Col-0 under each condition (*n*≥25). (C) Graph showing the ratios of root length at d 7 in the presence or absence of 4.5% sucrose, for the different *perk*, *kipk*, and *kcbp* mutant combinations. Ratios are root growth on ½ MS with 4.5% sucrose compared with ½ MS alone for each genotype. Asterisks represent statistically significant differences (*P*<0.05) when compared with ½ MS alone conditions for each genotype (*n*≥25). Col-0 and the single perk mutants (*perk8-1*, *perk-1*, and *perk10-1*) showed significant decreases in root growth on 4.5% sucrose plates. In contrast, significant increases in root growth on 4.5% sucrose plates were observed for the *perk8-1,9-1,10-1* triple mutant, the *kipk1-1,2-1* double mutant, the *kcbp-1* mutant, the *kipk1-1,2-1,kcbp-1* triple mutant, and the *perk8-1,9-1,10-1,kcbp-1* quadruple mutant. Seedlings for the other genotypes did not show any significant changes.

With the root being one of the tissues expressing the *PERK(8,9,10)-KIPK(1,2)-KCBP* set (Fig. 2), more focused root growth assays were conducted to search for shared mutant phenotypes. One of the root growth conditions tested was growing 7-d-old seedlings on different concentrations of sucrose (Hauser *et al.*, 1995) under a 16h light/8h dark cycle or a 24h light cycle (Supplementary Fig. S3 at *JXB* online.). The most significant changes were seen when the primary root lengths on plates without sucrose were compared with plates with 4.5% sucrose after 7 d of growth under a 24h light cycle (Fig. 4A, B, Supplementary Fig. S3, and Supplementary Table S2 at *JXB* online.). The *perk8-1,9-1,10-1* triple mutant, *kipk1-1,2-1* double mutant, and the *kcbp-1* mutant all displayed a significant increase in primary root length on 4.5% sucrose under 24h lighting when compared with Col-0 roots. This was not an osmotic effect, as the comparable concentration of mannitol produced no change in the root lengths of the mutants compared with Col-0. The increased primary root length of the mutants was also not seen on 0.5% sucrose, where the mutant roots tended to be a bit shorter than Col-0 (Fig. 4B).

When the primary root lengths from 4.5% sucrose plates were compared with plates without sucrose, a further trend was observed: whereas Col-0 displayed reduced growth on 4.5% sucrose, the multiple mutants had increased primary root lengths on 4.5% sucrose under 24h lighting (Supplementary Fig. S3, Supplementary Table S2). Thus, root growth in the absence or presence of 4.5% sucrose, under a 24h light cycle, was further tested across a number of different mutant combinations (Fig. 4C, Supplementary Table S2). The single *perk* mutants (*perk8-1*, *perk9-1*, and *perk10-1*) had a similar profile to Col-0 where there was decreased root length in the presence of 4.5% sucrose. However, the *perk* double mutants (*perk8-1,9-1*, *perk8-1,10-1*, and *perk9-1,10-1*) and the *kipk* single mutants (*kipk1-1* and *kipk1-2*) showed an intermediate phenotype where there was no significant change in primary root length under the two conditions. Finally, increased primary root lengths on 4.5% sucrose were observed for the *perk8-1,9-1,10-1* triple mutant, the *kipk1-1,2-1* double mutant, the *kcbp-1* mutant, the *kipk1-1,2-1,kcbp-1* triple mutant, and the *perk8-1,9-1,10-1,kcbp-1* quadruple mutant. Thus, while 4.5% sucrose had a somewhat inhibitory effect on Col-0 and the single *perk* mutant roots, the multiple *perk*, *kipk*, and *kcbp* mutants displayed increased root length on 4.5% sucrose under a 24h light cycle (Fig 4C and Supplementary Table S2).

### Overexpression of PERK10 in Col-0 and the *perk*, *kipk*, and *kcbp* mutants

**Fig. 5.**
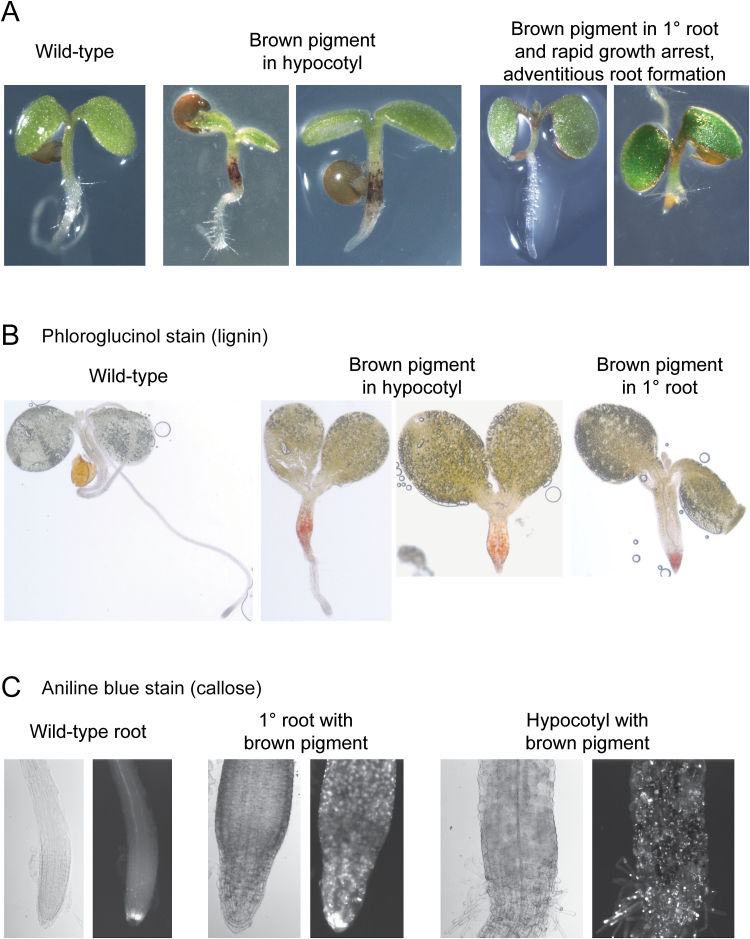
Phenotypes for the Col-0/*DEX::PERK10* seedlings. (A) Images of 5-d-old germinated seedlings. Seeds from primary Col-0/*DEX::PERK10* transformants were germinated in the presence of 10 µM DEX, and three different phenotypes were observed as shown. (B) Phloroglucinal staining for lignin in 5-d-old Col-0/*DEX::PERK10* seedlings. (C) Aniline blue staining for callose in 5-d-old Col-0/*DEX::PERK10* seedlings.

With the multiple *perk*, *kipk*, and *kcbp* loss-of-function mutants displaying increased root growth (under high sucrose and 24h lighting conditions), we also tested the opposite effect of overexpressing PERK10. We first attempted to overexpress PERK10 using the constitutive cauliflower mosaic virus 35S promoter but failed to recover any transgenic *Arabidopsis* seedlings. As a result, the *PERK10* cDNA was then expressed under the control of a DEX-inducible promoter in *Arabidopsis*, following treatment with DEX. Seeds from primary Col-0/*DEX::PERK10* transformants were germinated in the present of 10 µM DEX, and three different groups of phenotypes were observed: (i) seedlings that were wild type in appearance; (ii) seedlings with visible brown pigments in the hypocotyl; and (iii) seedlings with visible brown pigments in the root and rapid growth arrest of the primary root (these seedlings also produced adventitious roots) (Fig. 5A). When seeds from 20 independent Col-0/*DEX::PERK10* primary transformants were germinated in the presence of DEX, there was a range in the frequency of seedlings displaying brown pigments, with the strongest line showing 77% of the seedlings with visible brown pigments in the hypocotyl or the root (Supplementary Table S3 at *JXB* online.). These mutant phenotypes were never observed when transgenic Col-0 seeds with the DEX-only construct were tested (Supplementary Table S3). To test whether the brown pigmented tissues contained ectopic lignin (Rogers *et al.*, 2005), phloroglucinol staining was performed and produced the pink staining expected for lignin (Fig. 5B). The brown pigmented primary root and hypocotyl were also found to have ectopic callose deposits when stained with aniline blue (Fig. 5C). Thus, the overexpression of PERK10 in Col-0 seedlings resulted in some seedlings displaying root growth arrest with ectopic deposits of lignin and callose.

**Fig. 6.**
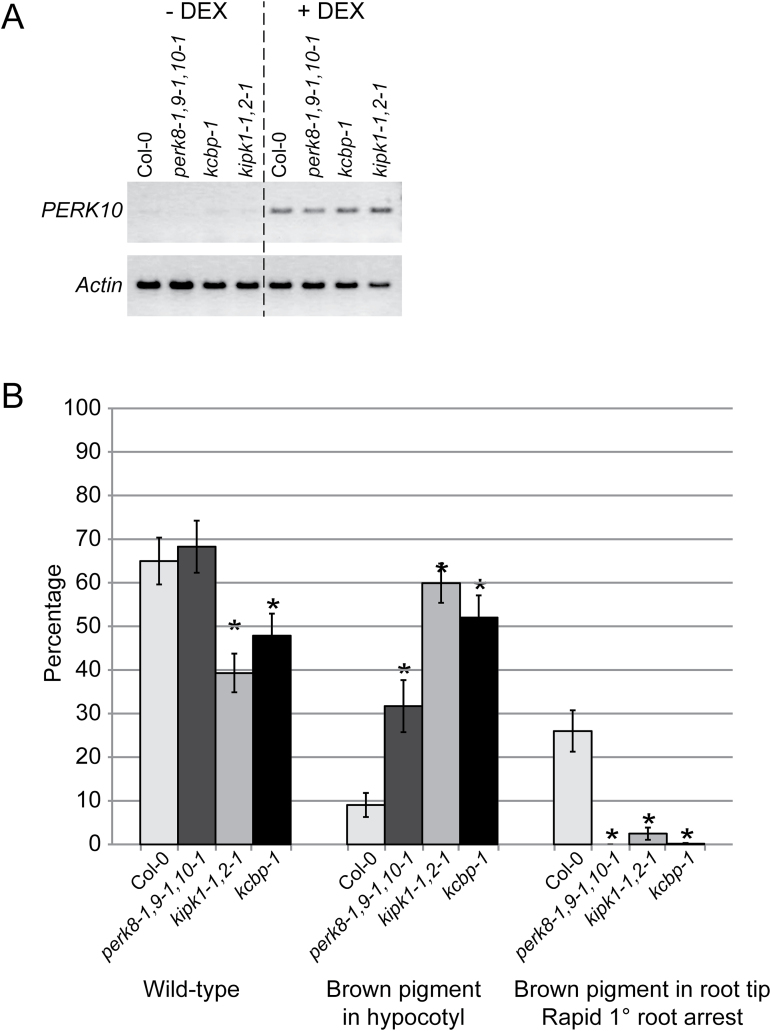
*DEX::PERK10* expression in the different mutant backgrounds. (A) RT-PCR analysis of PERK10 expression in 5-d-old *DEX::PERK10* seedlings from the different backgrounds, following no treatment (– DEX) or treatment with 10 µM DEX (+ DEX). Actin was used as a positive control for expression in all tissues. (B) Graph of the different phenotypes observed in 5-d-old *DEX::PERK10* seedlings. Seeds from primary transformants for Col-0/*DEX::PERK10*, *perk8-1,9-1,10-1*/*DEX::PERK10*, *kipk1-2,2-1*/*DEX::PERK10*, and *kcbp-1*/*DEX::PERK10* were germinated in the presence of 10 µM DEX and scored for the different phenotypes at d 5. For each genotype, seeds from 20 independent primary transformants were scored, and the mean was determined across the 20 lines (see Supplementary Table S3). Up to 25% of the seeds may be untransformed wild type, depending on the number of integrated transgenes, since the seeds were from primary transformants, and no transgene selection was applied.

The effects of overexpressing PERK10 was also tested in the different mutant backgrounds with the *DEX::PERK10* construct introduced into the *perk8-1,9-1,10-1* triple mutant, the *kipk1-1,2-1* double mutant, and the *kcbp-1* single mutant. RT-PCR analysis showed that expression of the *DEX::PERK10* construct was induced in all backgrounds with DEX treatment (Fig. 6A). For each background, seeds were collected from 20 independent primary transformants, germinated in the presence of DEX, and scored for the phenotypes resulting from PERK10 overexpression (Supplementary Table S3). Interestingly, while wild-type seedlings and seedlings with the brown pigmented hypocotyls were observed, very few to no seedlings were found to have the brown pigmented primary root with the rapid growth arrest (Supplementary Table S3). Figure 6B shows the percentage of seedlings in each category averaged over the 20 independent lines for Col-0 and the different mutant backgrounds. While Col-0/*DEX::PERK10* seedlings had a frequency of 26.01% with the brown pigmented primary root, 0% of the *perk8-1,9-1,10-1*/*DEX::PERK10* seedlings displayed this phenotype, and very low levels of the brown pigmented primary roots were detected in the *kipk1-1,2-1*/*DEX::PERK10* seedlings (2.45%) and the *kcbp-1*/*DEX::PERK10* seedlings (0.15%) (Fig. 6B and Supplementary Table S3). Interestingly, in all three mutant backgrounds, there was an increase in seedlings with brown pigmented hypocotyls in comparison with Col-0/*DEX::PERK10*. Nevertheless, in terms of root growth, the overexpressing PERK10 in the three different mutant backgrounds resulted in an attenuation of the brown pigmented primary root arrest phenotype observed in Col-0 (Fig. 6B).

## Discussion

In this study, we set out to identify *in planta* functions for the predicted PERK8, -9, and -10 receptor-like kinases and to identify their potential downstream signalling proteins. KIPK1 and -2 were found to be interactors of the cytosolic kinase domains from PERK8, -9, and -10, and as such, they represented candidate downstream signalling proteins. A GFP:KIPK1 was reported to be targeted to both the nucleus and cytoplasm in yeast (Zegzouti *et al.*, 2006), which would place KIPK1 in an overlapping compartment with the PERK8, -9, and -10 cytosolic domains for interactions. KIPK1 was identified previously as an interactor of KCBP (Day *et al.*, 2000), and we demonstrated here that this interaction is conserved for KIPK2. Future research will need to investigate the nature of these interactions *in vivo*, and the role of phosphorylation by the PERK(8,9,10) and KIPK(1,2) kinase domains during these interactions. To determine if the PERK(8,9,10)–KIPK(1,2) –KCBP **i**nteractions were biologically relevant and to determine if they were functioning as positive or negative regulators, T-DNA insertion mutants were screened for altered growth phenotypes. No obvious differences were apparent under standard growth conditions, and while *kcbp* mutants displayed a trichome phenotype (Oppenheimer *et al.*, 1997), this was not seen in the *perk* or *kipk* mutants. However, changes were observed on root growth assays under high sucrose and 24h lighting where the *perk8-1,9-1,10-1* triple mutant, the *kipk1-1,2-1* double mutant, and the *kcbp-1* mutant all displayed significant increases in primary root length compared with Col-0. Since all three mutant combinations displayed the same phenotype of increased root growth, this suggests that PERK(8,9,10) –KIPK(1,2)–KCBP are functioning in the same direction to negatively regulate root growth.

KIPK1 and -2 are members of the *Arabidopsis* AGC VIII kinases, which belong to the eukaryotic AGC serine/threonine protein kinase family, named after cAMP-dependent protein kinase, cGMP-dependent protein kinase, and protein kinase C (Bogre *et al.*, 2003; Zegzouti *et al.*, 2006). The *Arabidopsis* AGC VIII kinases are further divided into four subgroups, and KIPK1 and -2 belong to the AGC1 subgroup (reviewed by Rademacher and Offringa, 2012). Well-characterized *Arabidopsis* AGC VIII kinases include the AGC4 subgroup members, the PHOTOTROPIN1 and PHOTOTROPIN2 blue-light photoreceptors (Huala *et al.*, 1997; Jarillo *et al.*, 2001; Takemiya *et al.*, 2005). There are also the AGC3 subgroup members, PINOID, WAG1, and WAG2, which are proposed to regulate auxin fluxes by regulating the localization of PIN-FORMED (PIN) auxin efflux carriers (Christensen *et al.*, 2000; Santner and Watson, 2006; Dhonukshe *et al.*, 2010). Interestingly, the AGC1 subgroup includes D6 PROTEIN KINASE members that have also been implicated in regulating PIN proteins in relation to phototropic responses such as hypocotyl bending (Willige *et al.*, 2013). Finally, the AGC2 subgroup includes UNICORN functioning in planar growth regulation (Enugutti *et al.*, 2012) and OXIDATIVE SIGNAL INDUCIBLE1 required for signalling following oxidative bursts during root hair growth and basal pathogen resistance (Rentel *et al.*, 2004). One of the defining features of the AGC VIII kinases is the variable N-terminal domains that are thought to confer regulatory functions (Rademacher and Offringa, 2012). For KIPK1 and -2, this region is approximate 500 aa and displays 63% amino acid identity between the two proteins. The conservation of this region appears to be restricted to KIPK1 and -2 and not found in the other *Arabidopsis* AGC VIII kinases. There are no known predicted protein–protein interaction domains in this conserved N-terminal domain, and the only noticeable feature is an enrichment of serines. Our yeast two-hybrid data showed that the proximal N-terminal residues are required for KIPK1 and -2 to interact with the PERK cytosolic kinase domains while the distal N-terminal residues along with the C-terminal kinase domain are involved in the interaction with KCBP.

*Arabidopsis* KCBP was first isolated in a screen for proteins that bound calmodulin (CaM). The CaM-binding domain was mapped to the C-terminal end of KCBP and required Ca^2+^ for the interaction with CaM (Reddy *et al.*, 1996). The novel KCBP-interacting Ca^2+^-binding (KIC) protein was also demonstrated to bind to the C-terminal end of KCBP (Reddy *et al.*, 2004). KCBP is part of the kinesin-14 family, and kinesin-14 members are known to play roles in the organization of spindle poles and transporting cargo, and have C-terminal motor domains that drive minus-end-directed movement along microtubules (reviewed by Verhey and Hammond, 2009). As predicted, the KCBP motor domain binds microtubules, but this binding could be inhibited by both CaM and KIC in the presence of Ca^2+^ (Song *et al.*, 1997; Reddy *et al.*, 2004). Thus, CaM and KIC were proposed to be negative regulators of KCBP, and in keeping with this role, the overexpression of KIC resulted in trichomes with reduced branches, a phenotype found in the *kcbp*/*zwi* loss-of-function mutant (Oppenheimer *et al.*, 1997; Reddy *et al.*, 2004). With the possibility of KCBP also binding actin through the MyTH4 and the FERM domains (Reddy and Day 2000), Smith and Oppenheimer (2005) proposed a speculative model of KCBP interacting with microtubules and actin filaments to facilitate the transport of cell wall material from the Golgi to the plasma membrane for trichome branching (Smith and Oppenheimer 2005).

Thus, one possible cellular role of the PERK(8,9,10)–KIPK(1,2)–KCBP interactions in root growth may be to regulate cell expansion through the transport of cell wall material. We did not observe any differences in the lengths of expanded root cells (Supplementary Fig. S3B), and so the potential role of this pathway may be to regulate the rate of root elongation. Wuyts *et al.* (2011) found that when Col-0 seedlings were grown on 1% sucrose, the seedlings had longer roots and a faster rate of root elongation when compared with seedling roots grown with no sucrose. Therefore, the increased growth of the *perk8-1,9-1,10-1* triple mutant, the *kipk1-1,2-1* double mutant, and the *kcbp-1* mutant may be related to faster root elongation rates in the presence of 4.5% sucrose and 24h lighting. In contrast to the mutants, these conditions had an inhibitory effect on Col-0 roots (when compared with Col-0 seedling roots grown in the absence of sucrose), as reported previously (Hauser *et al.*, 1995). KCBP has also been found to be localized to microtubules in dividing tobacco BY-2 cells, suggesting a role during the cell cycle (Bowser and Reddy, 1997). This was further supported when antibodies to KCBP were injected into dividing stamen hair cells causing a disrupted phragmoplast formation and delayed cell division (Vos *et al.*, 2000). Thus, it is quite possible that the PERK(8,9,10)–KIPK(1,2)–KCBP interactions may also be influencing cell division rates during root growth.

In contrast to the mutant root phenotypes, the overexpression of *PERK10* led to a rapid arrest in primary root growth in wild-type Col-0 seedlings. The frequency of this phenotype was significantly reduced or absent in the transgenic *DEX::PERK10 perk8-1,9-1,10-1* triple mutant, the *kipk1-1,2-1* double mutant, and the *kcbp-1* mutant compared with transgenic Col-0/*DEX::PERK10* seedlings. This is what would be expected as the overactivation of this signalling pathway would be attenuated in these mutants compared with Col-0; that is, one would predict impairment of the signalling pathway downstream of PERK8, -9, and -10 for the *kipk1-1,2-1* double mutant and the *kcbp-1* mutant. For the transgenic *DEX::PERK10 perk8-1,9-1,10-1* triple mutant results, the simplest explanation is that the loss of *PERK8*, -*9*, and -*10* expression in the *perk8-1,9-1,10-1* triple mutant would decrease the total PERK activity following *DEX::PERK10* induction with DEX treatment, and as a result, primary root growth arrest is not observed. Thus, the occurrence of the primary root growth arrest phenotype in Col-0/*DEX::PERK10* seedlings, and reduced frequency of this phenotype in the transgenic *DEX::PERK10 perk8-1,9-1,10-1* triple mutant, the *kipk1-1,2-1* double mutant, and the *kcbp-1* mutant is consistent with the proposed role of PERK(8,9,10)–KIPK(1,2)–KCBP as negatively regulators of root growth. The second distinct phenotype observed with *PERK10* overexpression was germinating *DEX::PERK10* transgenic seedlings with brown pigment in the hypocotyl. This phenotype was enhanced in the transgenic *DEX::PERK10 perk8-1,9-1,10-1* triple mutant, the *kipk1-1,2-1* double mutant, and the *kcbp-1* mutant compared with transgenic Col-0/*DEX::PERK10* seedlings. It is unclear why there was an increased frequency of this hypocotyl brown pigment phenotype in the mutant backgrounds, but perhaps there are other unknown signalling components functioning in the hypocotyl to cause this enhanced phenotype with *PERK10* overexpression.

There is a general trend observed with other *perk* mutants of these genes acting as negative regulators of growth. A *perk13/rhs10* mutant had longer root hairs compared with wild-type, while overexpression of *PERK13/RHS10* in root hairs caused reduced root hair elongation and shorter root hairs (Won *et al.*, 2009). In addition, in the presence of ABA, *perk4* mutants displayed increased root elongation relative to wild-type Col-0 (Bai *et al.*, 2009). Among the complex traits present in the ectopic and antisense expression of *BnPERK1* in *Arabidopsis* were changes in hypocotyl length in dark-grown seedlings (Haffani *et al.*, 2006). Hypocotyls were significantly shorter with ectopic *BnPERK1* expression, while the antisense expression of *BnPERK1* leading to the suppression of *Arabidopsis PERK1* and -*3* resulted in longer hypocotyls (Haffani *et al.*, 2006). Thus, the overall theme suggests that PERKs act as negative regulators of plant growth, and under specific conditions or tissues, the loss of PERK function is associated with increased growth while increased PERK activity is associated with reduced growth. In the case of PERK8, -9, and -10, we propose that KIPK1 and -2 and KCBP function downstream to mediate their signalling responses.

## Supplementary data

Supplementary data are available at *JXB* online.

Supplementary Fig. S1. Amino acid sequence alignment of KIPK1 and KIPK2.

Supplementary Fig. S2. Phenotypes of *perk*, *kipk* and *kcbp* mutant plants.

Supplementary Fig. 3. Root growth of wild-type Col-0 and *perk8-1,9-1,10-1* triple mutant seedlings under different conditions.

Supplementary Table S1. Primers for RT-PCR analyses

Supplementary Table S2. Root lengths for the *perk*, *kipk*, and *kcbp* mutants under different conditions.

Supplementary Table S3. *DEX::PERK10* seedling phenotypes in Col-0 and the *perk*, *kipk*, and *kcbp* mutants.

Supplementary Data

## Abbreviations:

ABA: abscisic acid
CaM: calmodulin
DEX: dexamethasone
GFP: green fluorescent protein
KCBP: kinesin-like calmodulin-binding protein
KIC: KCBP-interacting Ca^2+^-binding
KIPK: KCBP-interacting protein kinase
NSP: nuclear shuttle protein
MS: Murashige and Skoog
PERK: proline-rich, extensin-like receptor-like kinase
RT-PCR: reverse transcription-PCR.
