# Supplementary material for: PERK–KIPK–KCBP signalling negatively regulates root growth in Arabidopsis thaliana
Source: J Exp Bot. 2014 Sep 26;66(1):71–83. doi: 10.1093/jxb/eru390 (PMC4265151; doi:10.1093/jxb/eru390)
Supplement: Supplementary Data [file supp_66_1_71__index.html]

PERK–KIPK–KCBP signalling negatively regulates root growth in Arabidopsis thaliana — PERK–KIPK–KCBP signalling negatively regulates root growth in Arabidopsis thaliana — Supplementary Data 

# PERK–KIPK–KCBP signalling negatively regulates root growth in *Arabidopsis thaliana*

## Supplementary Data

Data files

**Files in this Data Supplement:**

- Supplementary Data - Supplementary Data
