# Supplementary material for: PERK–KIPK–KCBP signalling negatively regulates root growth in Arabidopsis thaliana
Source: J Exp Bot. 2014 Sep 26;66(1):71–83. doi: 10.1093/jxb/eru390 (PMC4265151; doi:10.1093/jxb/eru390)
Supplement: Supplementary Data [file supp_eru390_jexbot127159_file001.pdf]

Supplementary Data

Humphrey T, Haasen K, Aldea-Brydges MG, Sun H, Zayed Y, Indriolo E, Goring DR

PERK - KIPK - KCBP signalling negatively regulates root growth in Arabidopsis thaliana.

```
KIPK1 MGSFAGACEIVEEKDDEVRLPKHSGRYGKSVMGSSSKDLVER  KQREYHGSLEYDIDMLFQSISVKPSTTRLMSSSFHHHETSASA 85
KIPK2 -E----S-------- A---A---S--CM-PL--- --MEQ-PALKSG-Q--M-----Q-----TI---PR-V-G-----L------ 85

KIPK1 GPSRTTSPSKRIASMKKP   GTPQSPRFVGLSDSVSLKQALRDRCISKASEMAAQKRLSKSAAASPRVSEADRIKSLYNQVSNEST 169
KIPK2 -T--S----N KGA----FPM---R--- --P---I--------L---------S---------------------T--R--L---A 170

KIPK1 SSRSGLVPVDKGKGSLVEIPLMPVNDKPSSSKSVPQRFE    DPSNPISEPSQAGTSFGLQG       VGNQTREIKLLHRSNKP 245
KIPK2 GKPG  L------ -----S-T--V-I----Q-----YDVLETE---F-------EILLHVL-NGSGIKT--YGML-TVS-CK---S 254

KIPK1 GSCLSSGSGDYEIELDENVASPSTHAFVEDDVIEIDKHVTSLPSHSSKKVNATELDKNISSSA VDSE       QKGKLDDAPNSG 324
KIPK2 --------------I---HT-- P-MVI--QLV-----------C-GS--DTE----S-V---R-K--PTALSSGL-----NF-G-- 340

KIPK1 TENGKTVRKVTRMIPRPK QPKKKILLKKKLKIGVVSATYPTKDDEEIVPSLDSSANQLLCQRCHCSLKSTSIDDNPPS   YTSSH 407
KIPK2 --KS-L-S----N-----PR-------------V-N---   -MV--VDT--EP--------K---AV----TENH---NTSH-TDK 424

KIPK1 NPKICTDSLSSVSN     KEAHQGSDENSSGSCNVSQSSEADIVIMKQDVSSSNNSGIGAMVEKETENPTSSEKFEFSLSSKDSLG 489
KIPK2 -VS-EA-QE-LA-PRLIRIVKCNKEASKG--D--E--D-G-- VIV---E--P--Y--K-DAD-QIRA--------D------N--- 510

KIPK1 DYSRSTSISEESNLSRFSCGNKPHMSMDVRWEAIKHIKVQYGSLGLRHFNLLKKLGCGDIGTVYLAELIGTNCLFAIKVMDNEFLAR 576
KIPK2 ---S---M-------------------------V--V-L-----------------------------V------------------ 597

KIPK1 RKKSPRAQAEREILKMLDHPFLPTLYAQFTSDNLSCLVMEYCPGGDLHVLRQKQLGRCFPEPAARFYVAEILLALEYLHMLGIIYRD 663
KIPK2 ---T-------A-------------------------------------------S---S---T------------------V---- 684

KIPK1 LKPENILVREDGHIMLTDFDLSLRCAVNPTLVRSNSPPGKDPARISGPYNTSNCIQPFCITEPSCQVSCFSPRLSSNQQQGRKPKRG 750
KIPK2 -------------------------------L--T---E-----M----S-------L-- ----R-P------L-T-ARNQ--RKP 770

KIPK1 DHLSKTQQHLSRSLPQLVAEPTEARSNSFVGTHEYLAPEIIKGEGHGAAVDWWTFGVLLYELLYGKTPFKGYNNDETLANVVLQNLK 837
KIPK2 KRPDLLT-QF -------------------------------------------------------------D-E---S---Y---- 856

KIPK1 FPDSPLVSFQAKDLIRGLLVKEPENRLGSEKGSVEIKRHPFFEGLNWALIRCAIPPELPDFYEYGGGPEAAADSPGGSNNRYLECKA 924
KIPK2 ------------E---R----D--S-------AA--------------------------I-DN-   ATE-T--E- --------- 939

KIPK1 IGDHLEFELF 934
KIPK2 ---------- 949
```

Fig. S1. Amino acid sequence alignment of KIPK1 and KIPK2.
Sequences were aligned using ClustalW2 (http://www.ebi.ac.uk/Tools/msa/clustalw2/).
Dashes are identical amino acids and spaces are gaps in the alignment.
The smallest yeast two-hybrid library clone identified for KIPK2 is double underlined: ______

The following conserved regions are highlighted in the alignment:
Serines - in conserved N-terminal region
Nuclear localization signal - predicted by NLStradamus (Nguyen Ba et al., 2009)
Nuclear export signal - predicted by NetNES (la Cour et al., 2004)
Serine/threonine protein kinase catalytic domain - predicted by SMART (Schultz et al., 1998)
S or T - phosphorylated serine or threonine in KIPK2 - from the PhosPhAt database (Heazlewood et al., 2008)
FELF - C-terminal PDK1-Interacting Fragment (PIF)-like domain (Zegzouti et al., 2006)

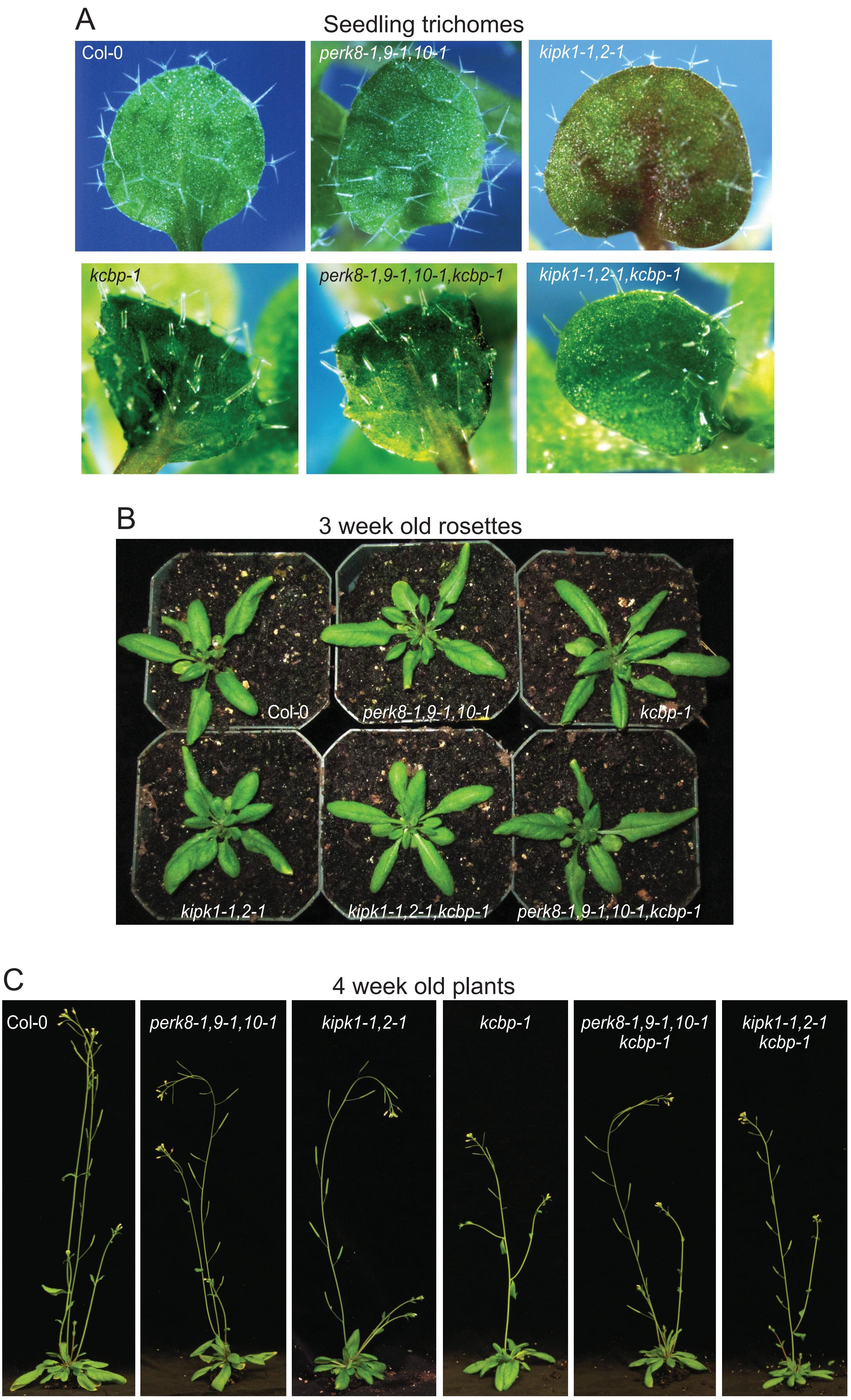

Fig. S2. Phenotypes of *perk*, *kipk* and *kcbp* mutant plants.
(A) Images of trichomes from seedlings at the 4- 6 leaf stage (grown on ½ MS plates).
(B) Images of rosettes from three-week old plants.
(C) Images of bolting four-week old plants.
Plants in both (B) and (C) were grown on soil in a 16 hour light/8 hour dark photoperiod at 22°C

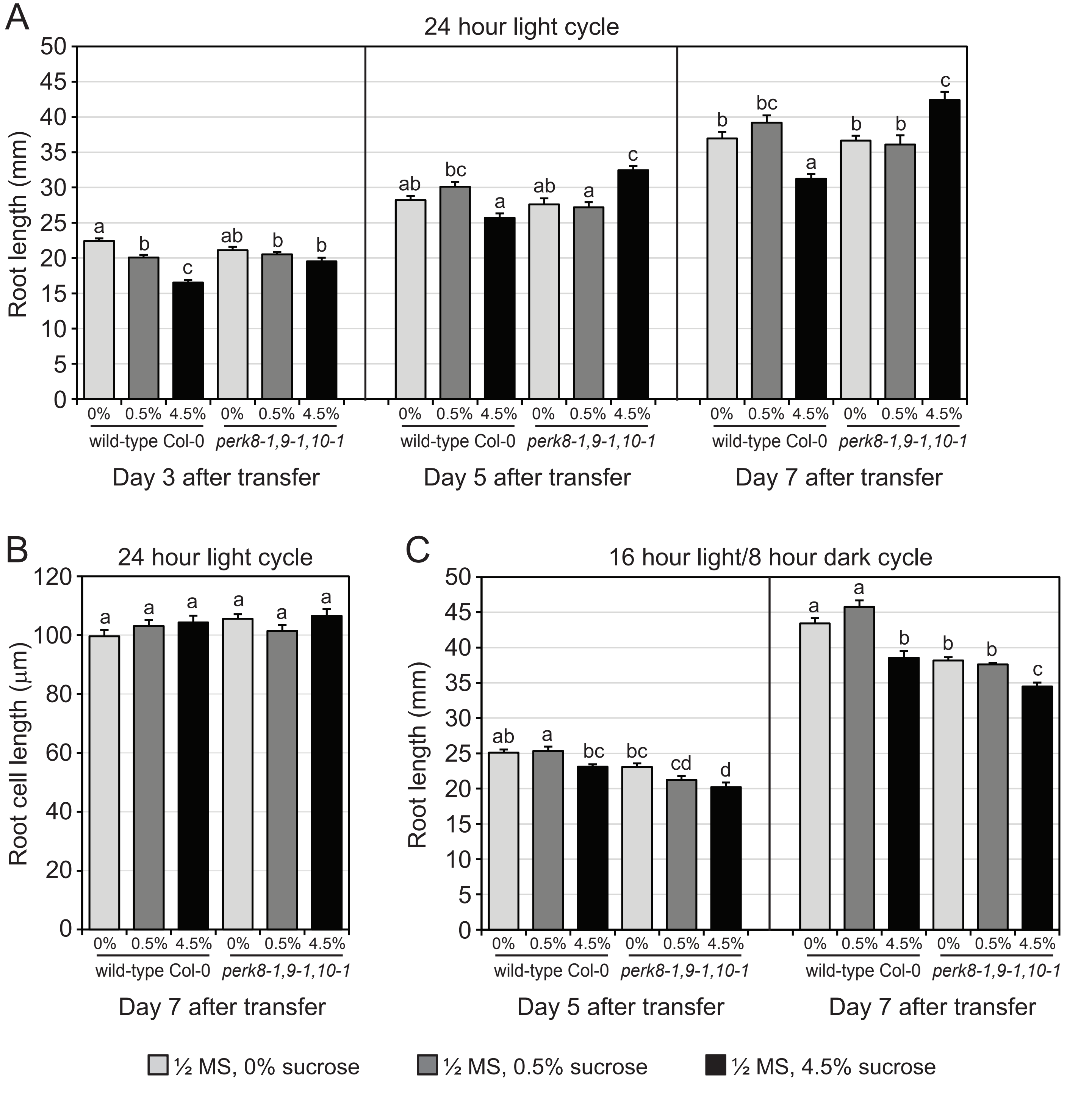

Fig. S3. Root growth of wild-type Col-0 and *perk8-1,9-1,10-1* triple mutant seedlings under different conditions.

(A) Graph showing root lengths at days 3, 5 and 7 after transfer. Seedlings were grown vertically under a 24 hour photoperiod on ½ MS plates with or without sucrose as indicated. n ≥ 25.

(B) Graph showing elongated root cell lengths at 7 days after transfer. Seedlings were grown vertically under a 24 hour photoperiod on ½ MS plates with or without sucrose as indicated. n = 50.

(C) Graph showing root lengths at days 5 and 7 after transfer. Seedlings were grown vertically under a 16 hour light/8 hour dark photoperiod on ½ MS plates with or without sucrose as indicated. n ≥ 25. The different letters represent means that are significantly different at P <0.05 for each timepoint (one-way ANOVA with Tukey-HSD post-hoc tests). Error bars = SE.

Table S1. Primers for RT-PCR Analyses				
Gene	Left Primer	Right Primer	Product Size Without Intron	Product Size With Intron
PERK8	GTCATCCCCGCATTATTCAC	GTCAAACACTTGGCTCTGTCCT	545	814
PERK9	TCTGTTCTAGACTGGGCAACAC	CGCCTAGTCTCATTCCGTTAGT	595	1007
PERK10	TACCATAAGCAGGGTACATCACC	TCTACCTAGCTTGGGATCTGCTA	577	1035
KIPK1	GAGGAGATCTTCATGTCCTCAGA	GGTCCACCACCATACTCATAGAA	856	934
KIPK2	CGCTGGAGTACTTACACATGCTT	CAGTGTCTCCTCATTGTCATAACC	530	No Intron
KCBP	TGATCTTCAAACCCAGTCTGC	ATCCACCAAGTCTTCTTCCTCA	445	862
ACTIN7	GGTGAGGATATTCAGCCACTT	TGTGAGATCCCGACCCGCAAGATC	555	735

Table S2. Root lengths for the *perk*, *kipk* and *kcbp* mutants under different conditions.

**Dataset #1.** 24 hr light, Day 7 root lengths (mm), Mean ± SE			
	½ MS, 0% sucrose	½ MS, 0.5% sucrose	½ MS, 4.5% sucrose
wild-type Col-0	36.80 ± 0.83	39.76 ± 0.61	34.94 ± 0.55
perk8-1,9-1,10-1	35.96 ± 0.74	34.80 ± 0.60	40.34 ± 0.99
kipk1-1,2-1	36.27 ± 1.00	36.93 ± 0.71	39.46 ± 0.67
kcbp-1	36.77 ± 0.79	37.05 ± 0.78	40.12 ± 0.49
kipk1-1,2-1, kcbp-1	36.63 ± 0.61	37.41 ± 0.64	40.46 ± 0.43
**Dataset #2.** 24 hr light, Day 7 root lengths (mm), Mean ± SE			
	130 mM Mannitol		
wild-type Col-0	26.40 ± 1.09		
perk8-1,9-1,10-1	23.90 ± 0.31		
kipk1-1,2-1	26.25 ± 1.03		
kcbp-1	26.28 ± 1.20		
**Dataset #3.** 24 hr light, Day 7 root lengths (mm), Mean ± SE			
	½ MS, 0% sucrose	½ MS, 0.5% sucrose	½ MS, 4.5% sucrose
wild-type Col-0	35.33 ± 0.43	37.04 ± 0.69	32.10 ± 0.48
perk8-1	34.23 ± 0.59	36.86 ± 0.60	32.07 ± 0.46
perk9-1	29.70 ± 0.36	30.27 ± 0.40	26.35 ± 0.37
perk10-1	30.90 ± 0.42	32.07 ± 0.34	28.42 ± 0.51
**Dataset #4.** 24 hr light, Day 7 root lengths (mm), Mean ± SE			
	½ MS, 0% sucrose	½ MS, 0.5% sucrose	½ MS, 4.5% sucrose
wild-type Col-0	37.00 ± 0.63	38.99 ± 0.64	34.42 ± 0.64
perk8-1,9-1	36.21 ± 0.77	37.01 ± 0.63	38.12 ± 0.70
perk8-1,10-1	36.95 ± 0.68	36.25 ± 0.52	37.22 ± 0.68
perk9-1,10-1	36.56 ± 0.61	35.27 ± 0.73	37.82 ± 0.67
**Dataset #5.** 24 hr light, Day 7 root lengths (mm), Mean ± SE			
	½ MS, 0% sucrose	½ MS, 0.5% sucrose	½ MS, 4.5% sucrose
wild-type Col-0	34.69 ± 0.44	35.20 ± 0.59	31.02 ± 0.51
kipk1-1	33.50 ± 0.57	32.00 ± 0.56	35.09 ± 0.81
kipk2-1	32.53 ± 0.74	30.65 ± 0.63	33.26 ± 0.69
perk8-1,9-1,10-1,kcbp-1	32.35 ± 0.64	33.25 ± 0.46	38.18 ± 0.81

The data in this table was used to calculate the ratios shown in Fig. 4.

Table S3. *DEX::PERK10* seedling phenotypes in Col-0 and the *perk* , *kipk* and *kcbp* mutants. Day 5 of the germination assay (% in each category)

	**Col-0/*DEX only***				**Col-0/*DEX::PERK10***				***perk8-1,9-1,10-1/DEX::PERK10***				***kipk1-2,2-1/DEX::PERK10***				***kcbp-1/DEX::PERK10***			
Primary transformants	N*	Wild-type**	Brown pigment in hypocotyl	Brown pigment in root tip; Rapid 1° root arrest	N*	Wild-type**	Brown pigment in hypocotyl	Brown pigment in root tip; Rapid 1° root arrest	N*	Wild-type**	Brown pigment in hypocotyl	Brown pigment in root tip; Rapid 1° root arrest	N*	Wild-type**	Brown pigment in hypocotyl	Brown pigment in root tip; Rapid 1° root arrest	N*	Wild-type**	Brown pigment in hypocotyl	Brown pigment in root tip; Rapid 1° root arrest
1	96	100	0	0	238	61	8	31	291	73	27	0	87	17	83	0	286	43	57	0
2	105	100	0	0	271	86	0	14	216	37	63	0	114	23	77	4	323	30	70	0
3	91	100	0	0	420	100	0	0	220	70	30	0	57	82	18	2	249	56	41	3
4	51	100	0	0	311	67	0	33	242	68	32	0	78	21	79	0	367	43	57	0
5	101	100	0	0	206	49.5	0.5	50	350	100	0	0	125	17	83	0	136	33	67	0
6	122	100	0	0	325	98.5	0.6	0.9	357	89	11	0	104	30	70	0	397	75	25	0
7	115	100	0	0	347	97	0	3	355	48	52	0	60	20	80	0	237	87	13	0
8	118	100	0	0	379	93	7	0	314	29	71	0	131	23	77	0	193	12	88	0
9	120	100	0	0	323	66	0	34	335	84	16	0	162	33	67	2	308	28	72	0
10	105	100	0	0	320	43	8	49	361	97	3	0	116	48	52	0	306	66	34	0
11	109	100	0	0	257	81	14	5	338	89	11	0	112	68	32	0	329	86	14	0
12	111	100	0	0	347	64	30	6	351	99.7	0.3	0	95	18	82	1	356	22	78	0
13	103	100	0	0	402	46	33	21	328	79	21	0	93	36	53	24	141	56	44	0
14	108	100	0	0	294	95.3	4	0.7	339	79	21	0	113	67	33	0	362	9	91	0
15	121	100	0	0	208	38	29	33	324	29	71	0	86	69	31	0	108	64	36	0
16	119	100	0	0	159	23	35	42	345	80	20	0	164	44	56	0	219	51	49	0
17	120	100	0	0	220	56.1	0.9	43	342	25	75	0	101	51	44	16	324	65	35	0
18	99	100	0	0	305	58	10	32	321	67	33	0	120	49	51	0	286	35	65	0
19	104	100	0	0	176	40	0	60	413	24	76	0	128	37	63	0	274	34	66	0
20	98	100	0	0	168	36.9	0.6	62.5	465	99	1	0	156	33	67	0	374	62	38	0
**Total:**	2116				5676				6607				2202				5575			
**Mean:**		100	0	0		64.965	9.03	26.005		68.285	31.715	0		39.3	59.9	2.45		47.85	52	0.15
**SE:**		0	0	0		5.359052	2.769648	4.73574		5.976107	5.976107	0		4.440898	4.493211	1.392603		5.066025	5.080924	0.15
**T-test:**										0.68149	0.001411	2.84E-06		0.000705	9.43E-12	2.7E-05		0.025761	6.58E-09	3.16E-06

*N = the number of germinated seeds screened from each independent primary transformant.

**Up to 25% of the seeds may be untransformed wild-type (depending on the # of integrated transgenes) since the seeds were from 1° transformants and no transgene selection was applied.
